# Structural, Microstructural and Compositional Changes of the AISI 314 Steel Used in the Sintering Furnace Belt Depending on the Operating Time

**DOI:** 10.3390/ma16237286

**Published:** 2023-11-23

**Authors:** Călin-Virgiliu Prica, Niculina Argentina Sechel, Miklos Tamas, Traian Florin Marinca, Florin Popa, Nurulla Orayev

**Affiliations:** 1 Materials Science and Engineering Department, Faculty of Materials and Environmental Engineering, Technical University of Cluj-Napoca, 103-106, Muncii Ave., 400641 Cluj-Napoca, Romania; niculina.sechel@stm.utcluj.ro (N.A.S.); traian.marinca@stm.utcluj.ro (T.F.M.); florin.popa@stm.utcluj.ro (F.P.); 2 Naposint Company, Rascruci, 407107 Cluj, Romania; miklos.tamas@naposint.ro (M.T.); nurullaorayev@gmail.com (N.O.)

**Keywords:** austenitic steel, sintering furnace belt, Cr depletion

## Abstract

The damage due to embrittlement of the sintering furnace belt and its replacement after a certain time of use represents a problem for the manufacturers of sintered parts. Finding out the reason for the damage could help to increase the duration of its operation. This research aimed to investigate the causes of embrittlement, considering both the temperatures and atmosphere of the sintering furnace to which the furnace belt is exposed during its operation. The furnace belt was made of AISI 314 stainless steel. Optical microscopy, scanning electron microscopy, combined with energy-dispersive X-ray analysis, X-ray diffraction and the Vickers hardness tests were used to analyze the microstructural, structural, compositional and hardness changes of the belt used for 45 weeks. Cr and Mn carbides, the oxides of Fe, Cr, Mn and Si were found to form at the edge of the furnace belt. The grains grew after 45 weeks of use, approximately 10 times, due to thermal cycles in an endothermic gas atmosphere to which the belt was exposed. Also, the hardness increased from 226 to 338 HV_0.05_, due to the formation of carbide and oxide-type compounds. All these results represent a starting point in optimizing the lifetime of the sintering furnace belt.

## 1. Introduction

The sintering process involves the heating of compacted parts at high temperature (mostly around 0.8 of the melting temperature), maintaining the parts at this temperature and cooling them down to ambient temperature [1]. Mostly, the compacted parts go through the stages mentioned above in the sintering furnace, placed on a metallic belt. Wire mesh belts are used to convey pressed parts through continuous sintering furnaces [2]. Also, the sintering process is carried out in specific atmospheres (depending on the type of powder used) in order to prevent oxidation of the parts [2,3]. For industrial applications, the most used atmosphere is endogas, since it is inexpensive and easily produced [4]. Endogas atmosphere contains H_2_, N_2_, and CO as the main components and small amounts of CO_2_ and H_2_O. The endogas is obtained by incomplete combustion of methane or propane gases [5]. It is known that the furnace belt is exposed both these gases, to some thermal cycles in different atmospheres during the sintering process and to mechanical stresses. These thermal cycles consist of repeated heating and cooling in the temperature range of 20–1120 °C. Usually, the sintering temperature of ferrous parts is in the 1100–1300 °C range [6,7]. The mechanical stresses occur due to the loading of the belt with parts. During operation at high temperatures, the furnace belt is subjected to complex interactions of temperature changes, oxidative and corrosive environmental attacks and mechanical stresses [8]. This interaction leads to embrittlement over time of the furnace belt, and thus, the furnace belt degradation is inevitable. Because of these reasons, the furnace belt is usually made by refractory steels. Refractory steels are richly alloyed in chromium, to which elements such as aluminium and silicon are added.

The most used refractory steels for a continuous sintering furnace are AISI 314 and 316 austenitic steels. Both steels are used in applications in which a good corrosion and oxidation resistance are required [9,10]. The oxidation resistance at high temperature of these stainless steels is due to a superficial layer of very dense and adherent oxides (Cr_2_O_3_, Al_2_O_3_ and SiO_2_ type) that separates the material from the aggressive environment [11,12,13,14]. When the austenitic steels work at high temperatures, intergranular corrosion may occur [15]. Due to the high temperature and the composition of the sintering atmosphere, during operation, the belt furnace material is also subject to cracking, oxidation, carburization, nitriding, etc. Chromium and manganese have a stronger tendency than Fe to form carbides [16]. 

Usually, the atmosphere of the sintering furnace in the high heating zone is a reducing one [17], which leads to the destruction of the protective Cr oxide layer. In the cooling zone of the sintering furnace, re-oxidation of the furnace belt occurs. Thus, during long-term use, the furnace belt is subjected to repeated oxidation and reduction cycles. To optimize the service life of the furnace belt, the steel from which it is made must be investigated after different operating times in order to observe the structural changes. Some researchers have studied these phenomena in the case of using sintering atmospheres rich in nitrogen highlighting the formation of Cr nitrides [18]. 

The aim of this work was the analysis of structural changes and the evolution of the hardness of the refractory AISI 314 steel from which the sintering furnace belt is made, what are the consequence of these changes on the embrittlement and how can the duration of its use be increased.

This research carried out together with the Naposint company, was very useful for the company in optimizing the operation time of the sintering furnace belt. The research will continue; the goal being the replacement of the sintering belt at longer time intervals, which will increase the productivity of the manufacture of ferrous sintered parts.

## 2. Experimental Methods

The samples were taken from a mesh belt of the Cremer continuous sintering furnace which is used for ferrous structural parts. The samples from the furnace belt were investigated at 0 (unused), 35 and 45 weeks. The usual parameters of sintering for ferrous parts are the following: sintering temperature—1120 °C, sintering time—30 min and the furnace atmosphere—endothermic gas (Table 1). 

The sintering furnace belts used in this study were manufactured from AISI 314 austenitic stainless steel wire by Bandas Metalicas CODINA Company (Torre de Claramunt, Spain). This company is a manufacturer of belts for sintering furnaces, producing a wide variety of types of belts. The belt used in this study was obtained from spirals to right and left joined together by a straight rod. Table 2 shows the belt’s chemical composition.

**Figure 1:**
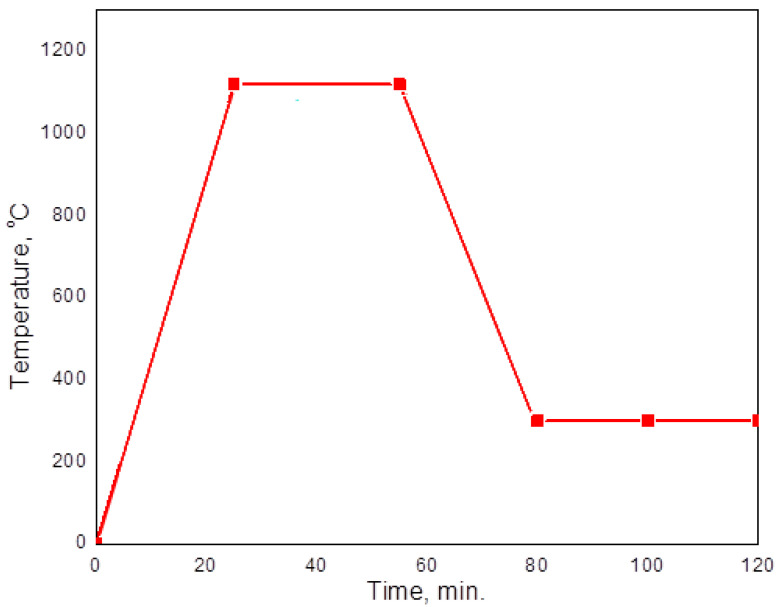
Continuous sintering furnace temperature versus time diagram.

The temperatures at which the furnace belt was exposed varied depending on the time according to the diagram in Figure 1. It is a specific sintering diagram for ferrous sintering parts. Three zones can be distinguished: heating zone (heating rate—35 °C/min), maintaining zone (1120 °C for 30 min) and cooling zone (cooling rate—30 °C/min up to 300 °C, from 300 °C to ambient temperature, cooling takes place in the air). The heating rates, maintaining time and cooling rates were given by the furnace belt speed.

**Figure 2:**
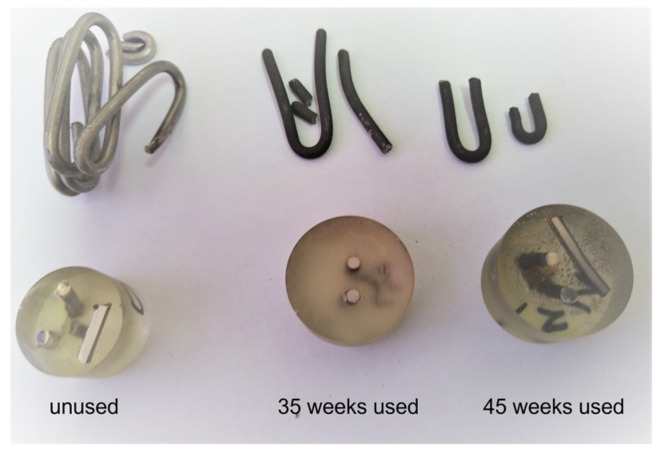
Photo images of samples from the furnace metallic belt (wire)—top and metallographic samples—bottom of the figure.

The microstructural analysis was investigated using optical microscopy (OM) and scanning electron microscopy (SEM) combined with energy dispersive X-ray local analysis (EDX). For metallographic analyses, samples were taken from the furnace belt (before use, after 35 and 45 weeks of use). The samples were then embedded in epoxy resin, sanded, polished, and then etching was performed for 30 s (by immersion) with Aqua Regia (Figure 2). 

Aqua Regia is a highly oxidizing mixture that is made by combining concentrated nitric acid (HNO_3_) and hydrochloric acid (HCl). The nitric acid/hydrochloric acid ratio was 1:3.

For optical microscopy, an Olympus inversed metallographic microscope with magnification up to 1000 times was used. A JEOL-JSM 5600 LV (Tokyo, Japan) scanning electron microscope (SEM) coupled with an energy-dispersive X-ray (EDX) spectrometer (Oxford Instruments UltimMax65, High Wycombe, UK) was used for the investigation of furnace belt microstructures and map distribution of elements. For the X-ray diffraction studies, an Inel Equinox 3000 diffractometer (INEL, Artenay, France) with Co radiation (λ_Co_ K_α_ = 0.17903 nm) was used. The diffraction patterns were recorded in the angular range 2θ = 20–100°. The acquisition was performed in one step in the entire 2 theta range, specific for the INEL diffractometer. The exposure time for each diffraction was 10 min. The hardness was determined with the Vickers hardness tester equipment manufactured by the Wolpert Group. The diamond indenter is pyramid-shaped, with a square base and an angle of 136 degrees between opposite faces. The intender loading was 0.05 kg. The space between indents was 0.5 mm, and the hold time was 15 s. HV_0.05_ median values were computed as the average of three tests.

## 3. Results and Discussions

**Figure 3:**
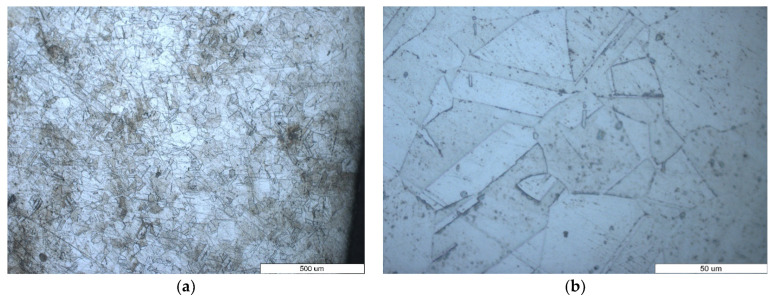
Optical images of the cross-section of unused furnace belt microstructure—(**a**) at the edge and (**b**) in the center of the sample (detailed).

The microstructures obtained by optical microscopy of an unused sintering furnace belt, manufactured by AISI 314 steel, are shown in Figure 3. It can be observed that the microstructure (on the wire transversal section) is austenitic, and the mean crystallite sizes is around 50 μm (Figure 3a). The chemical composition of AISI 314 steel (with high Ni content) and the specific polygonal shape of the grains is the clear proof of the austenitic structure. The grain boundaries are straight line, specific to austenite [19]. Grain size was estimated by visual comparison of several areas of metallographic samples. Also, it can be noted that the annealing twins were identified in the austenitic grains (Figure 3b). 

**Figure 4:**
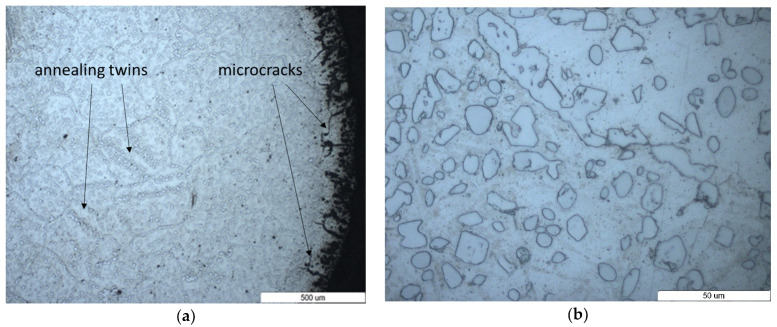
Optical images of the cross-section of furnace wire belt microstructures—35 weeks used (**a**) at the edge and (**b**) in the center.

**Figure 5:**
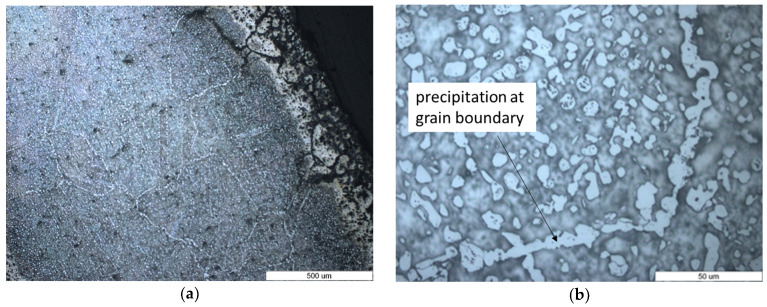
Optical images of the cross-section of furnace wire belt microstructures—45 weeks used (**a**) at the edge and (**b**) in the center (chemical etching—Aqua Regia).

Figure 4 shows the microstructures of the edge of the wire from which the belt was made (Figure 4a) and a detailed area of the center of the wire (Figure 4b) after 35 weeks of use. As compared with the microstructure of the unused wire belt, the growth of the austenite grains was observed, which corresponded to an overheating structure. This can be due to repeated heating at high temperatures up to 1120 °C, which is the sintering temperature. The austenite grains were 10 times higher than the grain of the unused wire belt (Figure 4a). Also, the discontinuous precipitates at the austenite grain boundary (Figure 4b) along the twins (Figure 4a) can be observed. At the edge of the sample, an area with microcracks or non-metallic compounds can be observed. One of the causes of microcracks is intracrystalline oxidation that occurs. The thickness of this area was around 200 μm. With increasing time of the belt use, after 45 weeks, it can be seen that the cracks or non-metallic compounds propagated inside the material at ever greater depths (Figure 5a). 

Also, the grains continued to grow, reaching dimensions of over 500 μm. It is also noticeable that the precipitates dispersed at the grain boundary became longer and continuous, no longer being concentrated in the twins (Figure 5b).

**Figure 6:**
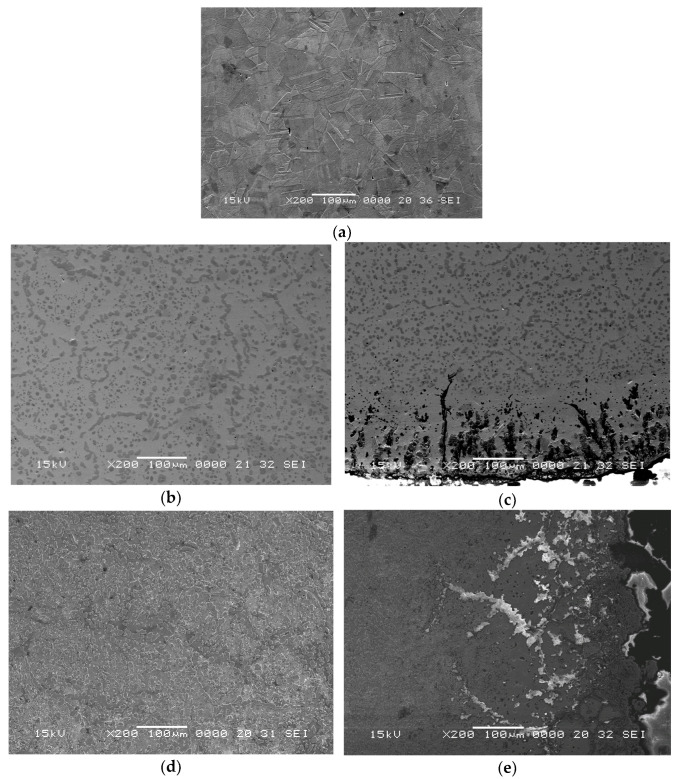
Microstructures (SEM images) of the cross-section of unused wire belt material—center of wire (**a**), after 35 weeks used—center (**b**) surface of the wire (**c**), and 45 weeks used—center (**d**) and surface of the wire (**e**).

The SEM images of unused, 35-weeks-used and 45-weeks-used materials from which the belt was made are shown in Figure 6.

The SEM images show that the structure of the AISI 314 steel from which the wire of the furnace band was made was austenite in the unused belt (Figure 6a), a fact that confirmed the results obtained by optical microscopy. After 35 and 45 weeks of use, in the center of the sample, the presence of the secondary phases at the austenite grain boundaries could be observed (Figure 6b,d). At the edge of the wire (Figure 6c,e) the microcracks with more than 100 μm length are shown. Also, the edge zone with defects reached about 500 μm after 45 weeks of use.

In order to highlight the nature of the secondary phases, the compositional analysis was carried out by SEM–EDX investigations.

**Figure 7:**
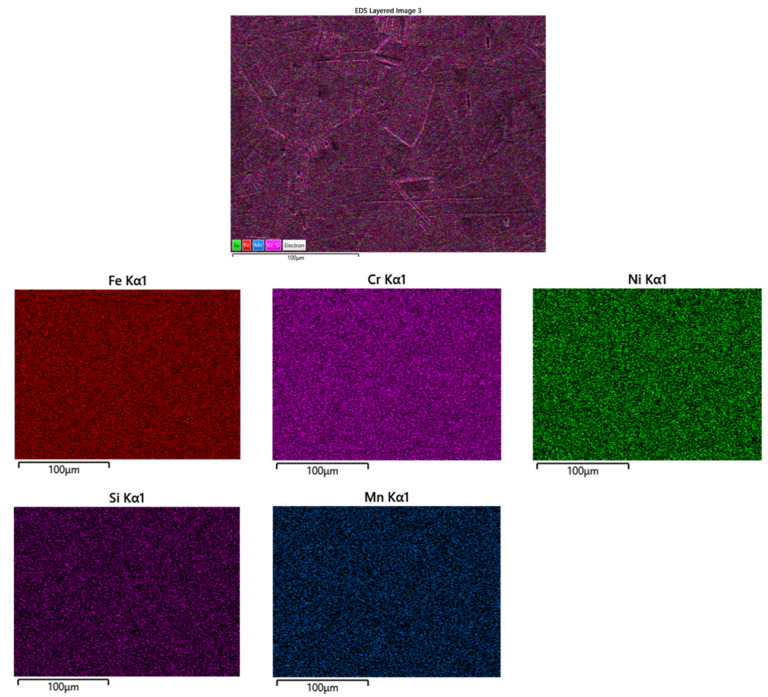
SEM–EDX maps distribution of elements of the cross-section in the center of the belt unused sample.

**Figure 8:**
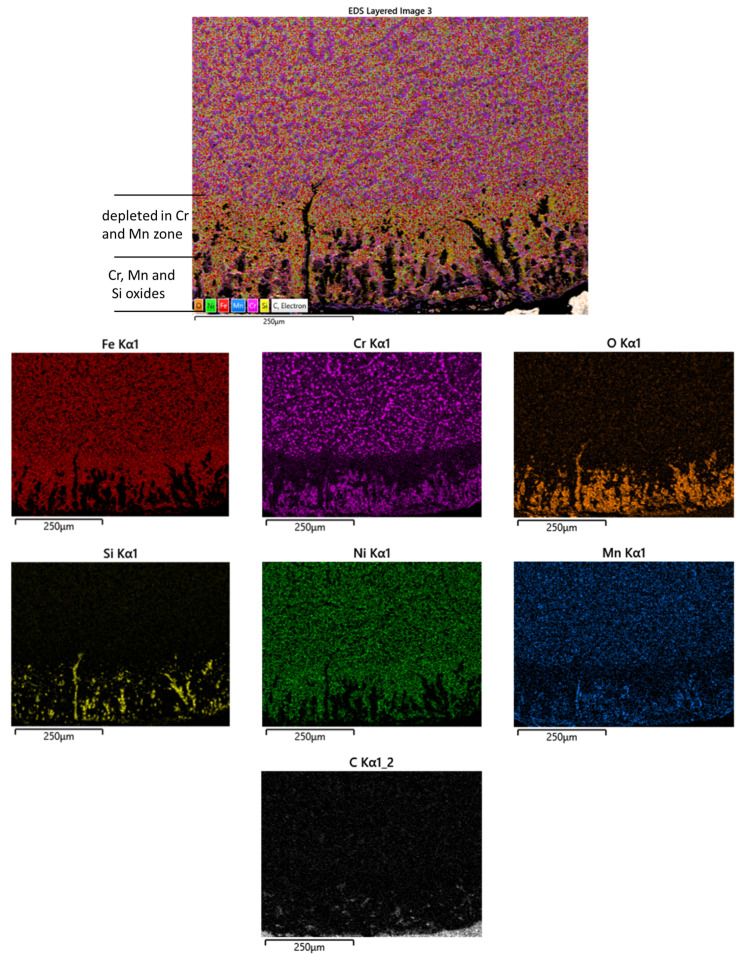
EDX maps distribution of elements of the cross-section of edge of the sample from the belt after 35 weeks of use.

The SEM combined with the EDX distribution map of the elements of the unused belt are shown in Figure 7. It can be observed that the elements from the composition of AISI 314 steel are evenly distributed in the analyzed area. In the case of the furnace belt after 35 and 45 weeks of use, the SEM and EDX analyses were undertaken both in the marginal area and in the central area of the wire. Figure 8 shows the SEM and EDX analysis of the edge of the sample from the belt after 35 weeks of use. 

In the edge area of the furnace belt, it can be observed that intergranular oxide penetrated inside the material up to 200 μm from the surface to the center of the sample. From the EDX elemental distribution maps, it was revealed that the intergranular oxides were of Cr, Si and Mn. They were formed because Oxygen from the furnace atmosphere, at the sintering temperature at which the belt operated for a long time, oxidized the belt surface, then diffused along the grain boundaries forming intergranular oxides. Also, an intermediate zone with a thickness of approximately 100 μm could be observed, in the immediate vicinity of the surface depleted in Cr and Mn. This phenomenon was also observed in other studies [20,21].

**Figure 9:**
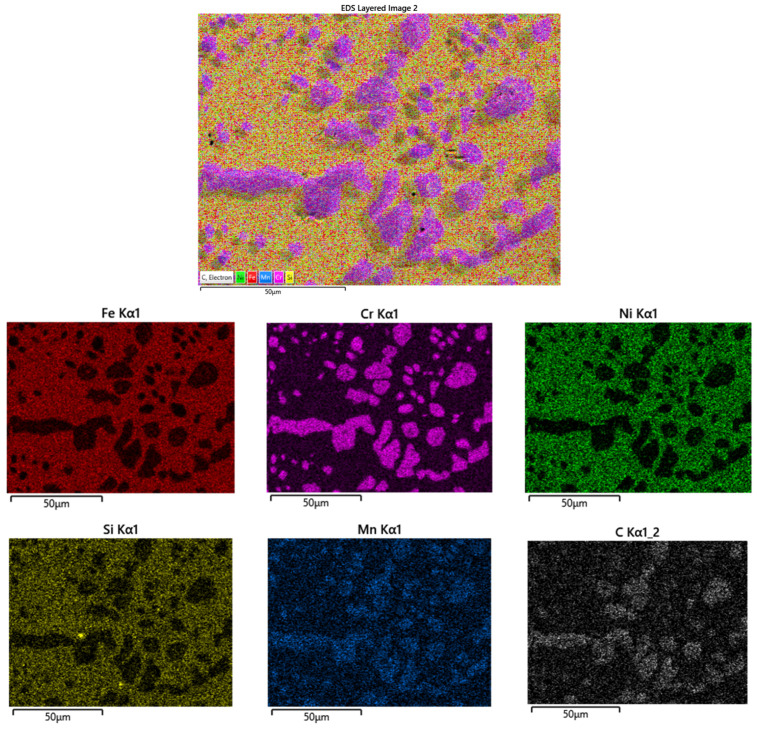
EDX maps of the distribution of elements of the cross-section, in the center of the sample from the belt after 35 weeks of use.

The EDX maps of the distribution of elements in the center of the belt sample used up to 35 weeks are shown in Figure 9.

It can be observed that the Fe, Ni and Si were uniformly distributed in the mass of the sample, except for certain areas where these elements were missing. On the other hand, these areas were rich in Cr, Mn and C, which indicated the presence of Cr and Mn carbides, discontinuously distributed at the grain boundary and inside them. The cause of the formation of these carbides is diffusion of C from the sintering atmosphere (in the carburizing zone of the furnace) along grain boundaries in the center of the sample. The transfer of carbon from the CO from the endogas to the steel surface takes place via the following reaction:

CO + H_2_ **→** C + H_2_O (1)

The carbide formation in the furnace belt structure leads to an increase in the hardness and of embrittlement. 

**Figure 10:**
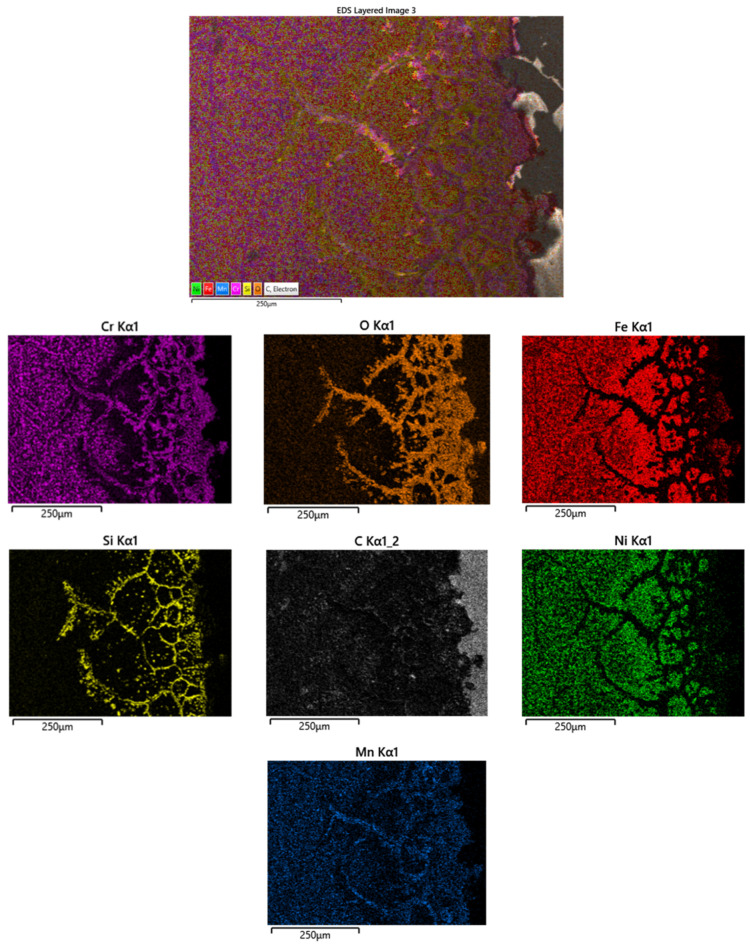
EDX maps distribution of element of the cross-section at the edge of the belt sample used up to 45 weeks.

Figure 10 shows the EDX map distribution of elements in the edge of the belt sample used for 45 weeks. It can be remarked that the affected area had a width of about 350 μm thickness. Similarly with the sample after 35 weeks of use, the Cr and Mn-depleted area with 250 μm thickness was observed. Both the damaged and the depleted areas were 100 μm larger in the case of the sample of belt used for 45 weeks.

In the 45 weeks sample, the oxides of Cr, Si and Mn were formed. Also, at the surface an Fe carbide layer with 100 μm thickness could be observed. 

**Figure 11:**
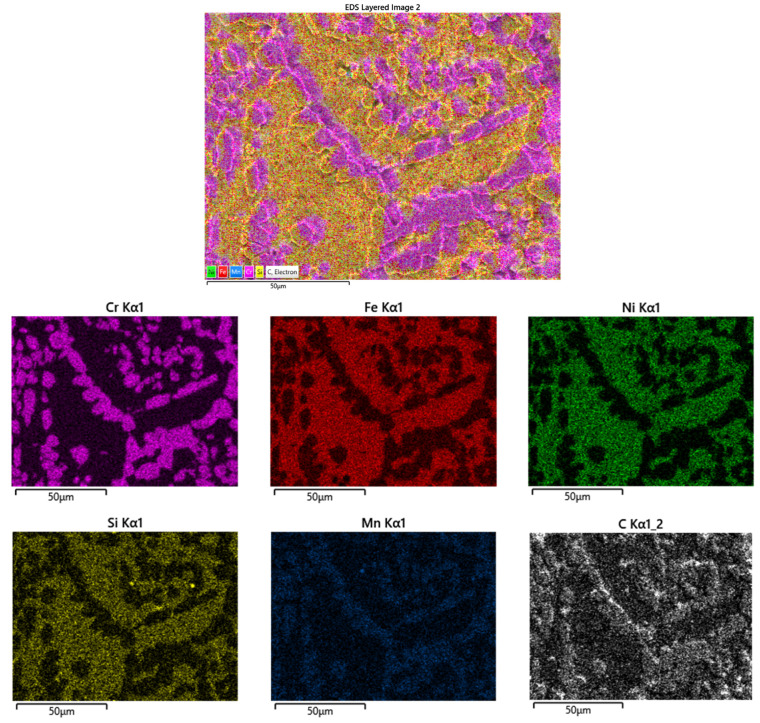
EDX maps distribution of elements of the cross-section, in the center of the belt sample used up to 45 weeks.

The EDX maps distribution of the elements in the center sample of the belt used for 45 weeks can be observed in Figure 11. Cr and Mn carbides are highlighted both as a continuous network at the grain boundary and uniformly distributed in the grains. 

**Figure 12:**
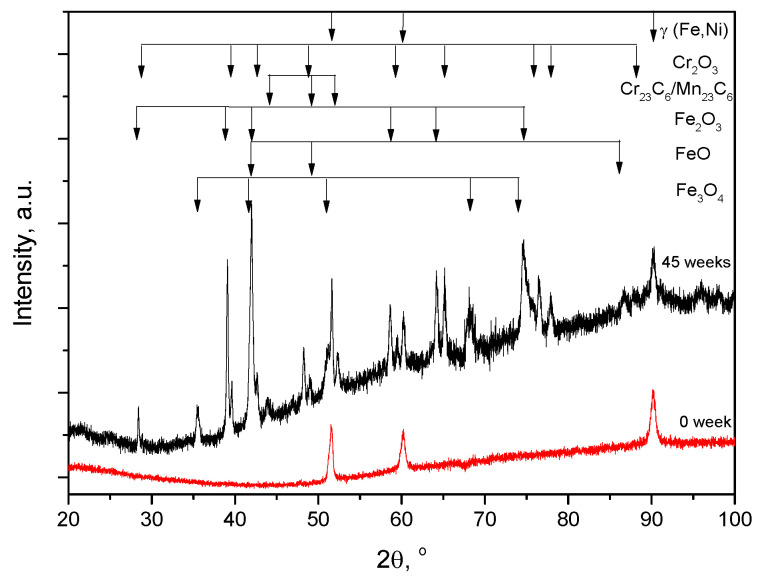
X-ray diffraction pattern of the belt—unused (red) and after 45 weeks of use (black).

In order to obtain a good identification of the carbides and oxides which were formed in the unused and 45-weeks-used belt furnace samples, XRD analyses were performed. The XRD patterns of furnace belt samples, unused and after 45 weeks of use are shown in Figure 12. In the XRD pattern of the unused sample, it can be observed that there were only the characteristic peaks of austenite (Fe, Ni solid solution) with FCC structure. This confirmed the results obtained through optical microscopy and the SEM–EDX analyses. In the XRD pattern of the furnace belt used for 45 weeks, in addition to the austenite (FCC) peaks, the maxima of carbides (Cr_23_C_6_ and Mn_23_C_6_) and iron and chromium oxides (FeO, Fe_3_O_4_, Fe_2_O_3_ and Cr_2_O_3_) can also be seen. The identification of the three iron oxides is normal since the intake of oxygen is increasing upon increasing the number of cycles and thus the oxidation becomes more and more prominent from the ferrous to ferrous–ferric and ferric oxides of iron. Due to the small amounts of Si and Mn (below the detection limit of the diffractometer), the maxima of their compounds did not appear in the diffraction pattern. The results are in accordance with OM and the SEM–EDX analyses. 

**Figure 13:**
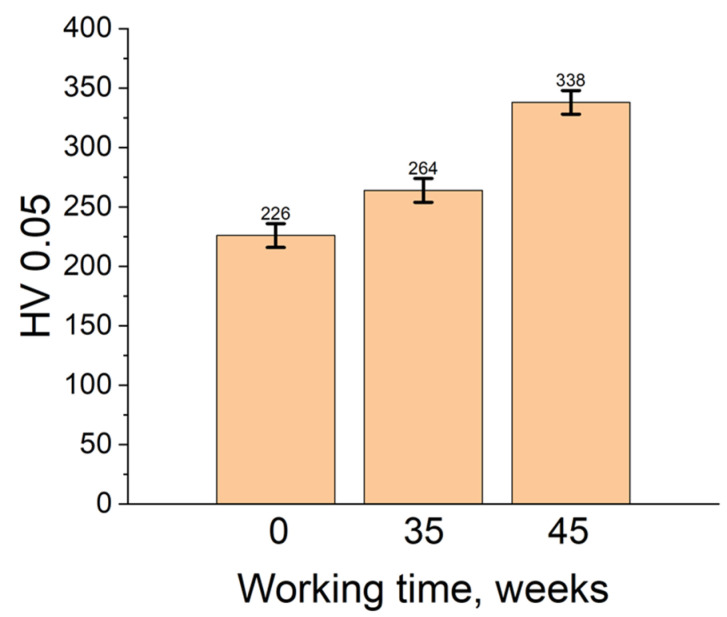
The Vickers hardness values of the belt used for 0, 35 and 45 weeks.

The carburizing effect on the furnace belt was also evaluated by the Vickers hardness measurements. The HV_0.05_ values of furnace belt unused, and after 35 and 45 weeks of use are shown in Figure 13.

The hardness measurements present values increasing with time of use for the furnace belt. So, the hardness HV 0.05 of the unused furnace belt was 226 and increased to the value of 264 after 35 weeks of use and reached 338 units after 45 weeks of use. This increase in hardness was the effect of the formation of Cr and Mn carbides and Cr, Mn and Si oxides in the edge area of the belt with increasing amounts over time as identified by SEM, EDX and X-ray diffraction. It is well known that the austenite phase is soft with high deformability. The austenitic structure is hardened due to the formation of the carbide network at the grain boundaries, oxides, and carbides at the surface of the belt material. All of these, combined with the occurrence of microcracks, leads to embrittlement of the structure of the furnace belt.

## 4. Conclusions

The investigations into the microstructures of sintering furnace belts leads to the following conclusions: the unused sample had an austenitic microstructure with grain sizes around 50 μm. Increasing the usage time to 35 weeks, some discontinuous precipitates at the grain boundary and along the twins appeared. Also, the austenite grain sizes increased up to 500 μm. The SEM–EDX analysis confirmed that the precipitates were Cr and Mn carbides. At the edge of the sample, the Cr, Mn and Si oxides were formed. After 45 weeks of use, in the microstructure of the belt, the carbide precipitates from the grain boundary became continuous, and the grains continued to grow over 500 μm. The grains growing was the effect of the overheating phenomena.

At the edge of both the samples, after 35 and 45 weeks of use an affected zone could be observed, containing Fe, Cr, Mn and Si oxides. This area size increased with the increase in the duration of use reaching 250 μm after 45 weeks of use. Also, in the immediate vicinity of the surface, a zone depleted in Cr and Mn could be identified. The Cr and Mn were diffused to the surface due to the higher affinity to oxygen compared to Fe and Ni.

The XRD pattern of the sample by the unused belt showed only the characteristic maxima of austenite (Fe-Ni solid solution) with an FCC structure. After 45 weeks of use, in the XRD pattern of the sample, in addition to the austenite (FCC) peaks, the maxima of carbides (Cr_23_C_6_, Mn_23_C_6_) and oxides (FeO, Fe_2_O_3_, Fe_3_O_4_ and Cr_2_O_3_) could also be seen.

The Vickers hardness measurements showed that the hardness of the furnace belt samples increased with the increase in the duration of use. Thus, the hardness grew from 226 HV0.05 for the unused sample to 338 HV0.05 for the sample used 45 weeks. The amount of Cr and Mn carbides and Cr, Mn and Si oxides which increased with the time of use of the furnace belt were the reasons for the hardness increasing.

The formation and growth of the layer of oxides and carbides in the surface layer of the sintering furnace belt, the formation of the carbide network at the grain boundaries and consequently, the hardening of the material, leads to its embrittlement.

## Figures and Tables

**Table 1:** The composition of the endothermic gas (sintering atmosphere) in which the belt works.

Endogas Composition	%
Hydrogen gas (H_2_)	40
Nitrogen gas (N_2_)	40
Carbon monoxide gas (CO)	19.5–19.8
Carbon dioxide (CO_2_)	0.2–0.5
Water vapor (H_2_O)	<0.1
Methane (CH_4_)	<0.1

**Table 2:** Chemical composition (%) of AISI 314 (X15CrNiSi25-21).

C	Si	Mn	Ni	P	S	Cr	N
Max. 0.2	1.5–2.5	Max. 2	19–22	Max. 0.45	Max. 0.015	24–26	Max. 0.11

## Data Availability

Data are contained within the article.
